# High-throughput optical sensing of peri-cellular oxygen in cardiac cells: system characterization, calibration, and testing

**DOI:** 10.3389/fbioe.2023.1214493

**Published:** 2023-06-16

**Authors:** Weizhen Li, David McLeod, John T. Ketzenberger, Grant Kowalik, Rebekah Russo, Zhenyu Li, Matthew W. Kay, Emilia Entcheva

**Affiliations:** Department of Biomedical Engineering, School of Engineering and Applied Science, The George Washington University, Washington, DC, United States

**Keywords:** optical oxygen sensors, peri-cellular oxygen, ruthenium oxygen sensors, human iPSC-CMs, cardiac fibroblasts, high-throughput plates

## Abstract

Human-induced pluripotent stem cell-derived cardiomyocytes (hiPSC-CMs) represent a scalable experimental model relevant to human physiology. Oxygen consumption of hiPSC-CMs has not been studied in high-throughput (HT) format plates used in pre-clinical studies. Here, we provide comprehensive characterization and validation of a system for HT long-term optical measurements of peri-cellular oxygen in cardiac syncytia (human iPSC-CM and human cardiac fibroblasts), grown in glass-bottom 96-well plates. Laser-cut oxygen sensors having a ruthenium dye and an oxygen-insensitive reference dye were used. Ratiometric measurements (409 nm excitation) reflected dynamic changes in oxygen, as validated with simultaneous Clark electrode measurements. Emission ratios (653 nm vs. 510 nm) were calibrated for percent oxygen using two-point calibration. Time-dependent changes in the Stern-Volmer parameter, *ksv*, were observed during the initial 40–90 min of incubation, likely temperature-related. Effects of pH on oxygen measurements were negligible in the pH range of 4–8, with a small ratio reduction for pH > 10. Time-dependent calibration was implemented, and light exposure time was optimized (0.6–0.8 s) for oxygen measurements inside an incubator. Peri-cellular oxygen dropped to levels <5% within 3–10 h for densely-plated hiPSC-CMs in glass-bottom 96-well plates. After the initial oxygen decrease, samples either settled to low steady-state or exhibited intermittent peri-cellular oxygen dynamics. Cardiac fibroblasts showed slower oxygen depletion and higher steady-state levels without oscillations, compared to hiPSC-CMs. Overall, the system has great utility for long-term HT monitoring of peri-cellular oxygen dynamics *in vitro* for tracking cellular oxygen consumption, metabolic perturbations, and characterization of the maturation of hiPSC-CMs.

## 1 Introduction

Oxygen is important for nearly all biological processes (Keeley and Mann, 2019). The function of aerobic cells relying on oxidative phosphorylation, such as cardiomyocytes, is highly dependent upon oxygen availability. Precise monitoring of oxygen levels in the immediate cell vicinity (peri-cellular oxygen) will provide invaluable insight into the metabolic state of the cells. Over the last two decades, human induced pluripotent stem cell derived cardiomyocytes (hiPSC-CMs) have emerged as a new scalable experimental model for cardiovascular research and translation (Burridge et al., 2016; Chen et al., 2016; Shi et al., 2017). The growth of these cells *in vitro* is being optimized for applications in regenerative medicine, drug development, cardiotoxicity testing, and other personalized medicine applications (Strauss and Blinova, 2017; Gintant et al., 2019; Yang et al., 2022). iPSC-CM oxygen consumption and metabolism are considered of key importance for their maturity (Rana et al., 2012; Sekine et al., 2014; Karbassi et al., 2020). For drug development and cardiotoxicity studies, human iPSC-CMs are typically grown in static culture using glass-bottom high-throughput format plates (96-well or 384-well plates). In such conditions, the only oxygen diffusion path is from the top, through the solution. Long-term studies of peri-cellular oxygen dynamics in human cardiac cells in such high-throughput plates are lacking, yet highly desirable.

Historically, oxygen sensing has evolved, starting with amperometric measurements using a Clark electrode (Clark, 1956; Severinghaus, 2004), where the electrochemical reduction of oxygen is registered by changes in electric current. This method still serves as the gold standard. Yet, it has limitations, especially for assessing oxygen dynamics in small spaces/volumes, including assessment of peri-cellular oxygen. This is due to the consumption/depletion of oxygen at the sensor during the measurement and the general difficulty in miniaturizing this type of sensor. Contactless optical methods present an alternative. A variety of fluorescent dyes have been developed to register very low (<5%) oxygen levels (Sandhu et al., 2017; Yoshihara et al., 2017), including near-infrared indicators for *in vivo* measurements (Kiyose et al., 2010). The limitations of such dyes include difficulty in providing a quantitative assessment and in covering a broader range of physiological values.

The Seahorse XF platform (Gerencser et al., 2009) is a high-throughput version of an optical oxygen measurement system. It has numerous applications in rigorous metabolic profiling of mammalian cells and isolated mitochondria (Rogers et al., 2011; Invernizzi et al., 2012; Divakaruni et al., 2014), including iPSC-CMs (Rana et al., 2012). It provides quantitative assessments of oxygen consumption rate (OCR) and extracellular acidification rate (ECAR), including in a 96-well format, as it offers oxygen and pH measurements through the optical sensors embedded in the tips of the fiber optics array. However, the Seahorse assay is applicable only to acute terminal measurements and therefore cannot be used for long-term tracking of peri-cellular oxygen dynamics.

Luminescence-based oxygen sensors, often combined with fiber optics, have been demonstrated to offer reliable oxygen tracking over time (Kostov and Rao, 1999; Kostov et al., 2000a; Liebsch et al., 2000; Wang and Wolfbeis, 2014). Their operation is based on dynamic oxygen quenching of fluorescence, as reflected in the Stern-Volmer relationship between oxygen concentration and fluorescence (Klimant et al., 1995). Quantitative optical oxygen sensing is typically done through life-time measurements (using frequency modulation) or through ratiometric intensity measurements with an oxygen-responsive dye (e.g. ruthenium-based) and a reference dye (Kostov et al., 1998; Kostov et al., 2000b; Wang and Wolfbeis, 2014). Scalability with such luminescence-based oxygen sensors has been achieved through improved matrix embedding of the dye and production of oxygen-sensing scaffolds for space-resolved measurements (Kellner et al., 2002; Wolff et al., 2019; Peniche Silva et al., 2020), as well as through advances in visualization with high spatiotemporal resolution (Tschiersch et al., 2012).

In this study we characterized and validated a high-throughput platform for longitudinal optical sensing of peri-cellular oxygen in human iPSC-CMs and human cardiac fibroblasts in 96-well format within a standard cell culture incubator. The system is based on the VisiSensTD oxygen imaging system (PreSens Precision Sensing GmbH, Germany) and our high throughput microfluidics-based uninterrupted cell culture perfusion system (HT-µUPS). Results demonstrate that the system provides accurate and reproducible long-term measurements of peri-cellular oxygen levels that will be valuable for studies of cellular oxygen consumption, metabolic perturbations, and characterization of the maturation of cultured iPSC-CMs.

## 2 Materials and methods

### 2.1 Optical oxygen sensors and peri-cellular oxygen imaging

**FIGURE 1:**
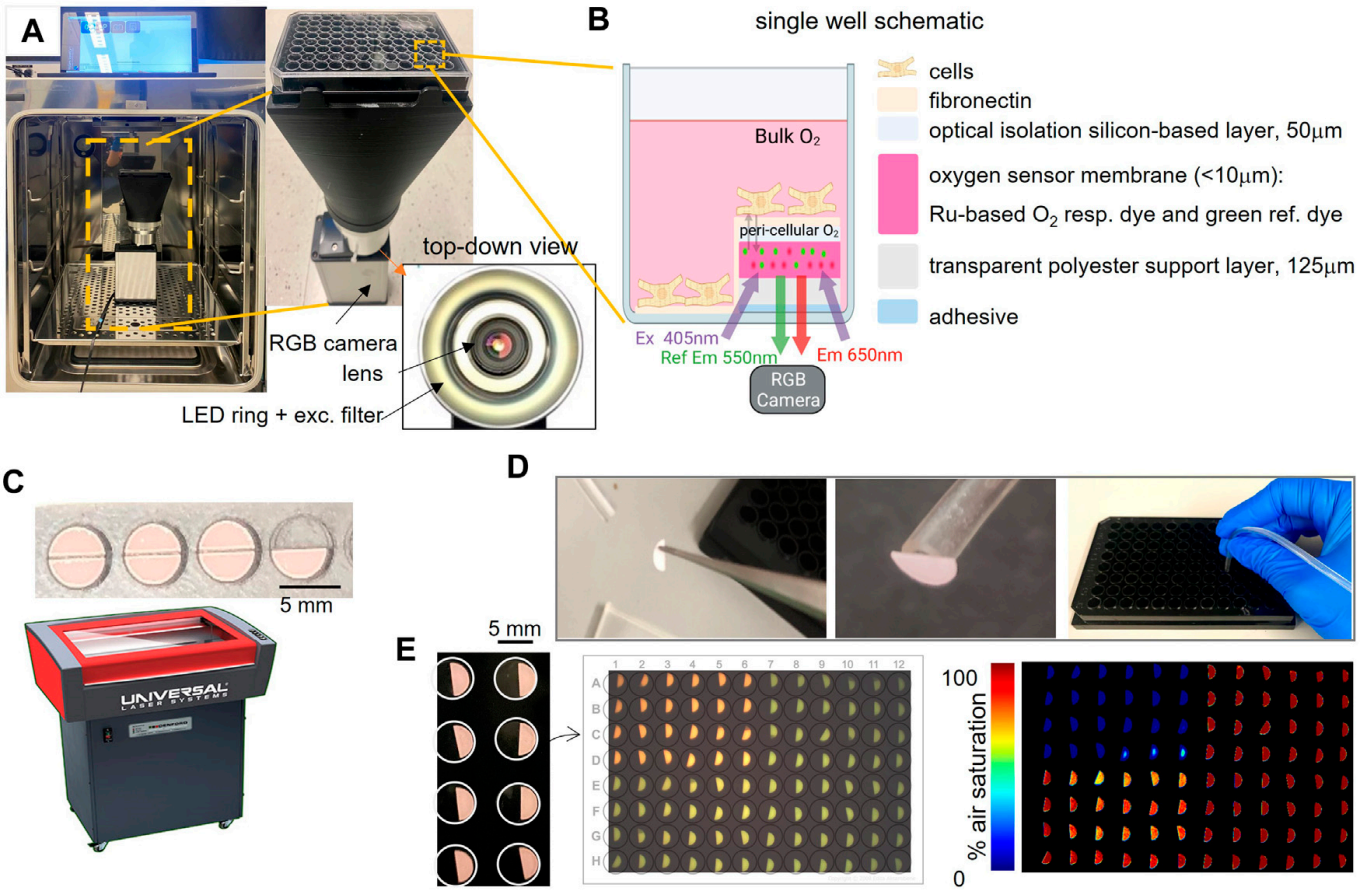
System and workflow for high-throughput optical measurements of peri-cellular oxygen. **(A)**. Incubator-deployed imaging system for 96-well plates, with an RGB camera, ring LED illuminator and bellows extension to fit a 96-well plate; inset shows top-down view with the lens and the LED ring with excitation filter. **(B)**. Schematic of a single well with an optical oxygen sensor composition and placement in half of the well. Peri-cellular oxygen is imaged ratiometrically through the glass bottom of the well plate; partial coverage allows for other parameters to be measured optically, e.g. voltage, calcium etc. **(C)**. Laser-cutting of semicircular oxygen sensor patches, 5 mm diameter. **(D)**. Mounting of the oxygen sensors: patches are detached from the adhesive backing and placed in wells using vacuum tubing. **(E)**. Images of the oxygen sensors in wells, raw optical readout and processed readings of oxygen. In the example, the upper left quadrant of the plate has been treated with oxygen-depleting Na_2_SO_3_.

Peri-cellular oxygen was measured using emission ratiometry. An integrated system comprised of an LED light source and an RGB camera (VisiSensTD, PreSens) imaged changes in the luminescence of optical oxygen sensors placed at the bottom of each well of a 96-well glass-bottom plate (Figure 1A). The plate was placed on top of the VisiSensTD system for continuous monitoring in a cell culture incubator. The oxygen sensor membrane (pink, Figure 1B) incorporated an oxygen-responsive ruthenium dye and an oxygen-insensitive reference dye. Blue light from the LEDs positioned around the camera lens excited the two dyes. The camera imaged the entire plate to acquire oxygen-dependent changes in the luminescence ratio of the two dyes within the sensor located in each well of the plate.

The oxygen sensors were semicircles that covered half the glass bottom of each well. They were laser-cut from a larger sheet (PreSens SF-RPSu4) that consisted of an oxygen sensitive layer, a polyester support layer, and a white optical isolation layer, which was placed onto a sacrificial acrylic sheet with the adhesive facing up (Figure 1C). Semicircular sensors were then laser-cut using a 30 W CO2 laser (Universal Laser Systems VLS 2.3) by placing the acrylic layer on the laser cutter bed and focusing the laser on the top of the acrylic layer. The laser cutting path was drawn in AutoCAD 2022 to cut semicircles with a radius of 2.5 mm using 15% maximum laser power and 10% maximum speed.

### 2.2 Oxygen sensor attachment and plate sterilization

Oxygen sensors were attached to the bottom of the wells of 96-well plates using sterile procedures inside a laminar flow hood. Sensors were lifted with tweezers to expose the white optical blocking layer while placing a suction tube (ID < 2 mm) against this layer to hold the sensor while lowering it into a well (Figure 1D). The suction in the tube was released once the adhesive layer attached to the glass. The process was repeated to place sensors in each well (Figure 1E). Before using the plate for a cell culture experiment, the wells were sterilized with 70% ethanol inside the sterile laminar flow fume hood. After the ethanol evaporated, each well was washed three times with 1x PBS, before coating with fibronectin. After an experiment, raw images acquired by the VisiSensTD system were processed using a two-point calibration to convert the RGB values for pixels that imaged each sensor to a percentage corresponding to the peri-cellular oxygen level. An example raw luminescence image showing the optical sensors in a 96-well plate and the processed oxygen reading is illustrated in Figure 1E.

### 2.3 Oxygen sensor spectral characterization

**FIGURE 2:**
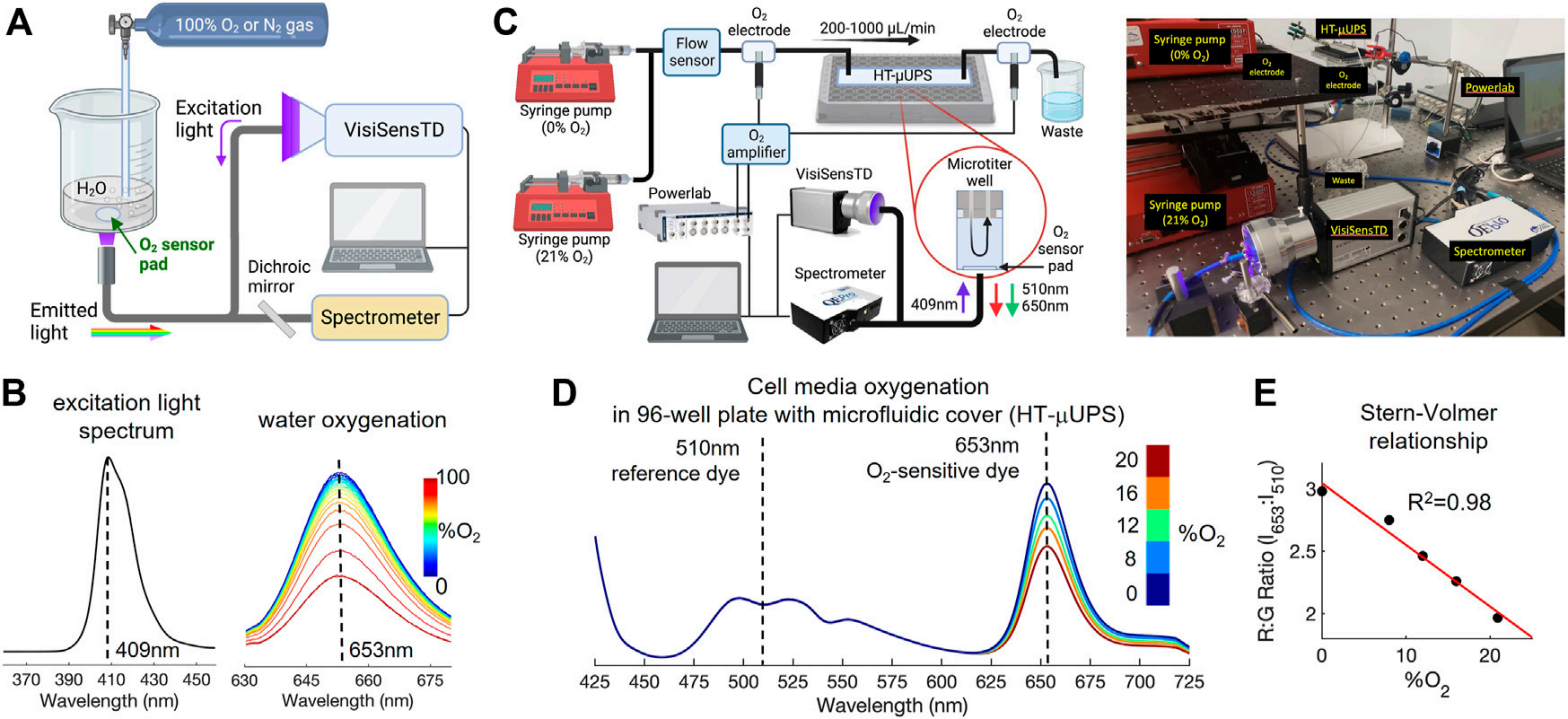
Spectral characterization of the optical system for peri-cellular oxygen imaging and confirmation of an inverse Stern-Volmer relationship. **(A)**. Schematic of the setup used to characterize oxygen-sensitive responses in a beaker of water. **(B)**. Spectral results in water: excitation light emission is at 409 nm; oxygen-sensitive emission peak is at 653 nm, with emission decreasing as O_2_ concentration increases. **(C)**. Schematic and photo of the setup for spectral characterization of oxygen responses in a 96-well microplate with a specialized microfluidic cover (our HT-μUPS system). The oxygen sensor pad is at the bottom of one well. **(D)**. Spectral data in cell culture media, showing oxygen-dependent spectral shifts at 653nm, consistent with results from water shown in panel **(B)**. **(E)**. An inverse Stern-Volmer relationship was derived from the ratios (intensity at 653/intensity at 510), measured from the spectra shown in panel **(D)**, which is linear for the low oxygen range considered (<20%).

Spectra for the full range of aqueous oxygen concentration were first measured by bubbling a beaker of water with 100% O_2_ gas followed by bubbling with 100% N_2_ gas (Figure 2A). A sensor attached to the bottom of the beaker was illuminated with excitation light from the VisiSenseTD system and the spectrum of emitted light was acquired once every second using a spectrometer (Ocean Optics QE-Pro, Figure 2B).

Spectra for oxygen concentrations that are typical for cell cultures were acquired using a similar approach and our high throughput microfluidics-based uninterrupted perfusion system (HT-µUPS) cover for a 96-well plate (Wei et al., 2020) (Figure 2C). Nitrogen-bubbled cell culture media (0% O_2_) and media equilibrated in room air (21% O_2_) were loaded into two separate syringes that were each placed in one of two syringe pumps (New Era 1600 × 2). Media from each syringe flowed through a sensor (Sensirion SLI-2000) that measured the flow rate as media moved through our HT-µUPS cover to perfuse the wells of the plate (Figure 2C). Flow-through Clark electrodes (Microelectrodes Inc. MI-730) incorporated into the tubing before and after the plate measured the media oxygen concentration at those positions. The system flow rate was maintained at 200 ml/min while the flow rates of the two syringe pumps were varied to achieve mixtures of 0, 8, 12, 16, and 20 percent O_2_, which were confirmed by the Clark electrodes. The same spectrometer and fiber optic cable setup as in Figure 2A was used to acquire the luminescence spectrum of a sensor within one well once every second.

Spectra collected during each characterization were plotted together to visualize changes in spectral bands corresponding to the oxygen-insensitive reference dye (centered at 510 nm, “green”) and the oxygen-responsive ruthenium dye (centered at 653nm, “red”, Figure 2D). Values at the peak wavelengths for red and green luminescence (Figure 2D, dashed lines) were used to construct the inverse Stern-Volmer relation in Figure 2E. For this relationship, the ratio of red to green emission was plotted against each oxygen concentration measured by the Clark electrode placed before the plate. A linear fit of ratio *versus* oxygen concentration was used due to the small oxygen concentration range of 0%–20% that corresponds to typical cell culture conditions.

### 2.4 Oxygen-impermeant HT-µUPS cover and temporal characterization

The responsiveness of the oxygen sensing system to changes in media oxygen concentration and flow rate was characterized using 96-well plates and a new oxygen-impermeant version of the HT-µUPS cover described in our previous work (Wei et al., 2020). This cover was assembled in two components, one soft PDMS sealing gasket and one acrylic perfusion base that was CNC-milled from cast acrylic (McMaster-Carr 8560K354) and thermally bonded using a heat press (Rosineer Grip Twist) at 130°C for 3 h. The sealing gasket was fabricated by pouring a mixture of Sylgard 184 (Dow) and Dragonskin 10 (Smooth-On) in a 1:2 ratio into an acrylic mold. Finally, an inlet and outlet were tapped (10–32 thread) for each well and fitted with stainless steel 10–32 barb-to-thread connectors (Pneumadyne).

**FIGURE 3:**
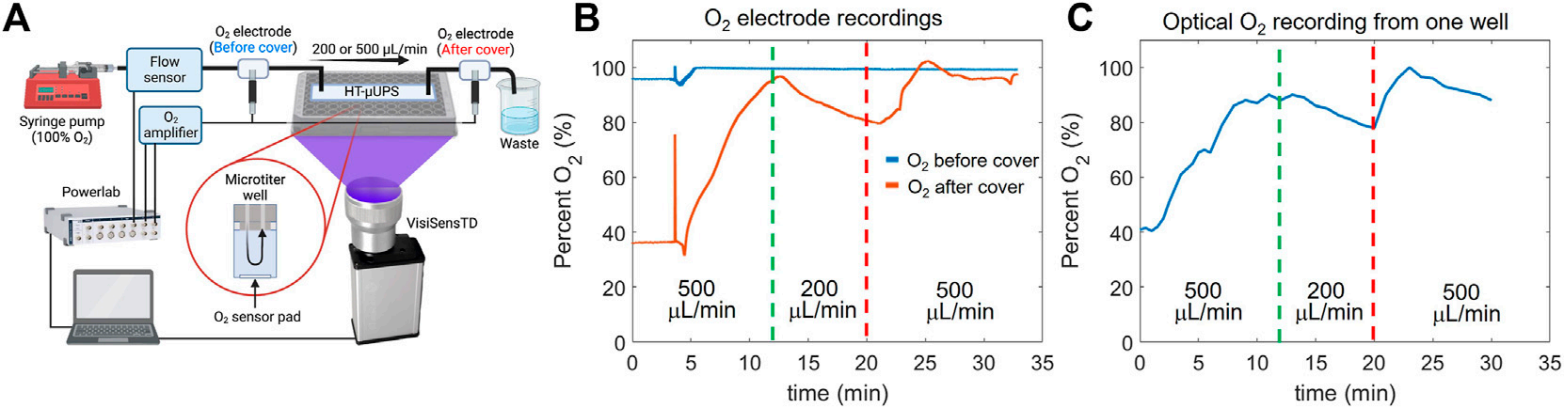
Validation of optical oxygen measurements with an electrochemical Clark electrode. **(A)**. Schematic of the setup used to perform the validation. The HT-μUPS was with an acrylic microfluidic cover. **(B)**. Measurements with the Clark electrodes positioned before and after the cover. Perfusion flow was varied to alter the oxygenation of the 96-well plate (see vertical lines). **(C)**. Simultaneous measurements with the optical system reading the optical oxygen sensor within the microwell.

The sensor response within the perfusion system HT-µUPS was validated using the setup in Figure 3A. Clark electrodes were placed before and after the cover. An optical oxygen sensor was placed at the bottom of the leftmost well of the cover. The Clark electrodes and VisiSensTD system were calibrated with water at 100% O_2_ and 0% O_2_. Water bubbled with 100% O_2_ was loaded into a syringe pump and perfused through the cover. Flow rate was measured using a flow meter (Sensirion SLI-2000) and varied between 500 ml/min and 200 ml/min to determine the system’s response to changes in flow.

### 2.5 Two-point oxygen calibration

A two-point calibration converted the ratio of red to green luminescence intensity imaged from the sensors into percentage of air saturation similar to (Peniche Silva et al., 2020). The terms ‘Cal0’ and ‘Cal100’ were used in system characterization experiments for 0% and 100% air saturation, and oxygen saturation levels ranging from 0% to 18.6% were displayed in cell experiments (the equilibrium oxygen partial pressure in a humidified 37°C, 5% CO_2_ incubator is 18.6%).

Two types of culture media, CDI iCell Cardiomyocytes^2^ maintenance medium (Fujifilm CDI) and cardiac fibroblasts growth medium (Cell Applications, Inc.), were used. The air-saturated medium (Cal100) was medium straight from the bottle, warmed to around 37°C. And the 0% air-saturated medium (Cal0) was medium with 5% completely dissolved Na_2_SO_3_ and placed in the 37°C water bath for 30 min. In testing pH’s influence on oxygen readout, the media were adjusted with NaOH or HCL to pH 10 or pH 4.

### 2.6 Cell plating and peri-cellular oxygen monitoring

Human iPSC-derived cardiomyocytes (iCell Cardiomyocytes^2^ CMC-100-012-001 from a female Caucasian donor) from Fujifilm Cellular Dynamics International (CDI), and human cardiac fibroblasts, CF (Cell Applications, Inc.) were thawed according to manufacturer’s instructions. Cells were plated (50,000 cells per well) in the wells of a 96-well glass-bottom plate containing half-moon shaped oxygen sensors, that have been sterilized and fibronectin-coated (at 50 μg/ml). Culture medium exchange was done every 48 h. In some experiments, hypoxia was induced on day five after plating by filling the wells to the top with culture medium and sealing them with oxygen-impermeable tape before readout in the VisiSense system.

The VisiSenseTD system was temperature-equilibrated in the cell culture incubator at least an hour before the start of measurements. For the whole plate oxygen monitoring in human iPSC-CMs, the peri-cellular oxygen measurements started 5 h after the cell plating, after the switch from the cell plating medium to the cell maintenance medium. And for the hiPSC-CMs and cardiac fibroblast hypoxia comparison experiment, oxygen monitoring started right after the hypoxic condition was established. Oxygen recordings were set to continue for 24–48 h, with a 10 min sampling interval and 0.8 s exposure time.

### 2.7 Cell visualization

Cells grown on the non-transparent oxygen sensors were visualized using an upright Leica TCS SP8 microscope with a ×25 water immersion objective (for this imaging, sensors were attached to the glass bottom 35 mm dishes). Cell imaging of the glass-bottom portion of the 96 wells was performed using an inverted confocal microscope (Zeiss LSM 710) or inverted Nikon Eclipse Ti2 microscope. Samples were formaldehyde-fixed, permeabilized and labeled as described previously (Li et al., 2020; Wei et al., 2020). Nuclei were labeled with Hoechst (H3570, Thermo-Fisher Scientific), the cytoskeleton was labeled either for F-actin using Alexa-488 phalloidin (A12379, Thermo-Fisher Scientific) or using α-actinin antibody (A7811, Millipore-Sigma), and some samples were genetically modified to express the optogenetic actuator Channelrhodopsin-2 with a fluorescent reporter eYFP.

### 2.8 Oxygen data analysis

Peri-cellular oxygen readings were acquired as PNG images and then analyzed through VisiSensVS software by selecting regions of interest in each image. Measured ratios were calibrated to percentage oxygen readings using two-point calibration with 5% Na_2_SO_3_ and upon saturation with ambient air. Time-dependent calibration files were applied in the first 2 h of recording, considering the temperature-induced changes in transferring the plate from room temperature operation to the 37°C humidified incubator. Whole-plate data normalization was applied by identifying the maximum and minimum ratio readouts from the whole plate throughout the whole period of recording. A scale factor was calculated by setting the maximum oxygen reading as 18.6%, the equilibrium oxygen concentration in a cell culture incubator.

### 2.9 Statistical analysis

To quantify the influence of exposure time on the ratio of red to green luminescence intensity, the mean and standard deviation (mean ± SD) of ratios from a 96-well plate were computed and displayed. The spatial variation of the ratios across a plate was assessed for 0.8 s of exposure time. For time-dependent calibration and for the assessment of the effect of pH on oxygen measurements, the change in the ratio of red to green luminescence intensity over time was calculated and displayed as (mean ± SD).

## 3 Results

### 3.1 Mechanism of optical peri-cellular oxygen measurements

Optical peri-cellular oxygen measurements were based on dynamic oxygen quenching of a ruthenium dye embedded in the sensor patches on top of which cells were grown. For quantitative ratiometric readout, red and green luminescence intensity ratios were converted to percentage of oxygen saturation (pO2) using an adapted Stern-Volmer equation:

$$\frac{R_{o}}{R}={\left(\left(\frac{A}{1+ksv*pO2}\right)+\left(1-A\right)\right)}^{-1}$$ (1)

R is the measured luminescence ratio, R_0_ is the luminescence ratio at 0% O_2_, *ksv* is the Stern-Volmer constant indicating the efficiency of oxygen quenching, and A is 0.82, a parameter for the non-linearity of the sensing material (Peniche Silva et al., 2020). The ksv was computed using the two-point calibration described in the Methods, and a time-dependent ksv was applied at the beginning of the recordings to account for temperature changes. Eq. 1 was linear for the limited range of oxygenation, that is, typical for cell cultures (<20%, Figure 2E). However, when measured over a full range of 0%–100% oxygen concentration, the inverse Stern-Volmer is expected to follow a decaying exponential function (Wang et al., 2010).

### 3.2 High-throughput compatibility

The semicircular oxygen sensors covered half of each well in a 96-well glass bottom plate (Figures 1C, D). This feature enabled multiparametric optical high-throughput measurements from the other half of each well. Such measurements include cellular action potentials, intracellular calcium transients, and contractility (Klimas et al., 2020; Heinson et al., 2023; Liu et al., 2023). Laser cutting of the sensors was quick and reproducible (Figure 1E), where more than 96 half-moon sensors could be cut in less than 10 min. Subsequent attachment of the sensors in each well of a 96 well plate could be completed in less than 1 hour. Laser cutting had a negligible effect on sensor performance and provided sufficient sensor area for good ratiometric measurements after selecting a region of interest from each sensor. We found that it is essential to keep the pre-cut sensors in the dark and to use them within 6 months of laser cutting for best results. Sterilization of the sensors with ethanol for cellular experiments did not affect their performance.

### 3.3 System characterization and validation

The RGB images of the entire plate (1280 × 1024 pixels, 24bit) acquired by the VisiSensTD system provided sufficient contrast with approximately 1000 pixels per sensor, and 300 to 700 pixels per sensor region of interest (Figure 1E, middle). After calibration, the pseudocolor images of the plate clearly denoted differences in oxygenation between wells. In the example shown in Figure 1E, right, the quadrants of the plate were conditioned with 0% air-saturated PBS (top left), 100% air-saturated PBS (top right), room air (bottom left), and 100% air-saturated PBS with pH = 4 (bottom right). We found that solution pH had negligible influence on calibrated oxygen sensor values. The oxygen sensors also reacted differently in air than when submerged in solution.

Spectral characterization of the optical oxygen sensors and validation of the oxygen measurements with Clark electrodes confirmed that small changes in peri-cellular oxygen concentration could be accurately measured in 96-well plates (Figure 2 and Figure 3). The Presens oxygen sensors (SF-RPsSU4) had a peak emission of 653 nm upon excitation with 409 nm light, and this emission decreased in response to increased oxygen concentration (Figure 2B), demonstrating dynamic ruthenium fluorescence quenching by oxygen (Wang and Wolfbeis, 2014; Wolff et al., 2019). Increased fluorescence in the green band (485–570 nm) was also measured from the sensors upon illumination with 409 nm light (Figure 2D), with a midpoint of 510 nm. The intensity of this band did not change when oxygen concentration increased, corresponding to the oxygen insensitive reference dye that Presens has added to the sensor material. These controlled spectral measurements in a 96-well plate using our HT-µUPS system confirmed adequate spectral sensitivity of the sensors for the range of peri-cellular oxygen that is expected for cell cultures (<18.6%). The relationship of the ratio of red to green emission intensity was linear (Figure 2E), as predicted by Eq. 1.

A recent study cross-validated VisiSensTD peri-cellular oxygen measurements using a fiber-optics oxygen microelectrode (Peniche Silva et al., 2020). In contrast, we used in-line Clark electrodes to validate the measurement of peri-cellular oxygen using laser-cut semicircular sensors, the VisiSensTD system, and physiological flow rates through our HT-µUPS system in a 96-well plate (Figure 3A). Our system demonstrated appropriate responsiveness to changes in media oxygen concentration and flow. During a 30 min recording, media flow began at 500 ml/min, was reduced to 200 ml/min, and then restored to 500 ml/min (Figure 3A). Oxygen values from the Clark electrode positioned at the inlet of the plate indicated that media oxygenation was near 100% and remained constant (Figure 3B). Oxygen values from the Clark electrode positioned after the plate aligned with values measured optically from the sensor at the bottom of the first well. The diameter and length of tubing and the time of the peak oxygen value (85%) measured at the bottom of the well (Figure 3C, green line) corresponded to the flow rate of 500 ml/min. The drop in oxygenation at 200 ml/min indicated a loss of oxygen as the media traveled more slowly through the connection at the entrance of the HT-µUPS cover.

### 3.4 Optimal exposure time

**FIGURE 4:**
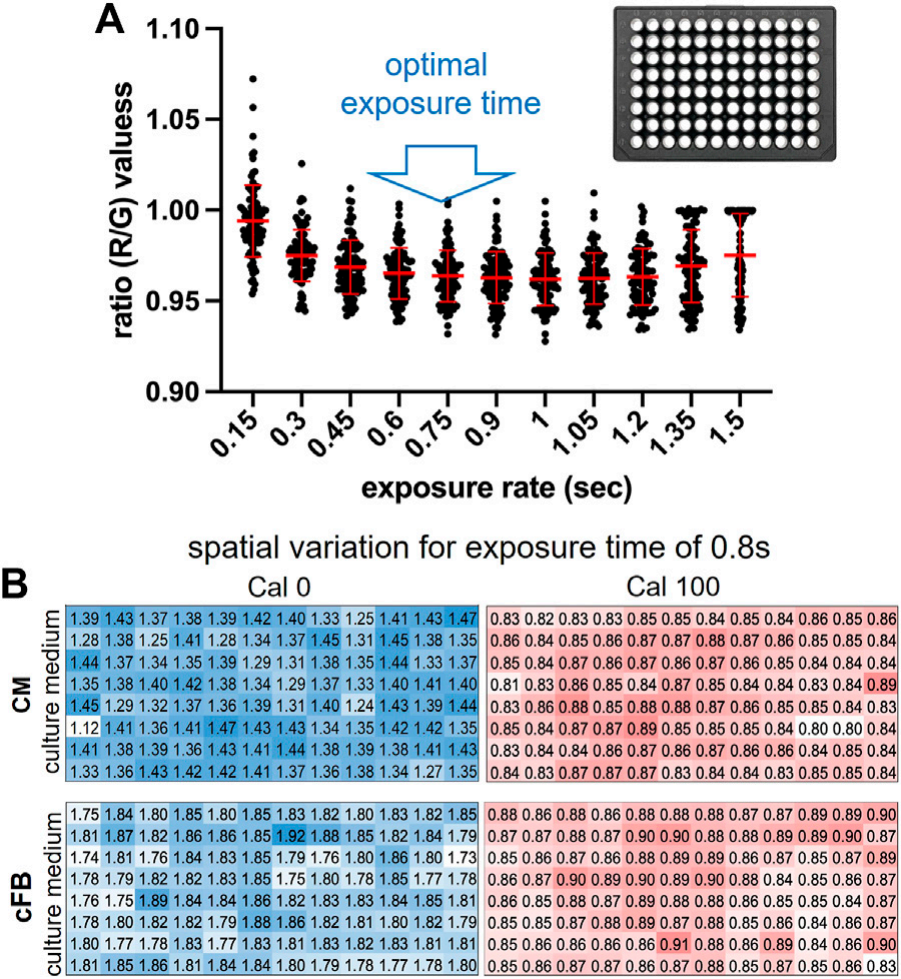
Influence of exposure time on ratio readouts and their spatial spread in a 96-well plate. **(A)**. Oxygen readout (ratio values) spread across the 96-well plate as exposure time varies between 0.15 and 1.5 s, dot plots and mean ± SD. To minimize the spread, exposure was chosen in the 0.4–0.9 s range. **(B)**. Spatial variation across the 96-well plate with 0% oxygen (Cal0), blue, and air saturated (Cal100) oxygen for culture medium used to grow human cardiac myocytes, CM (top), and human cardiac fibroblasts, cFB (bottom). 5% Na_2_SO_3_ in the medium was used for 0% oxygen calibration; exposure time set to 0.8 s.

Excitation light was not uniformly distributed across the bottom of 96-well plates. This increased the spatial variance (well-to-well differences) of red:green emission ratios from the sensors for short excitation light exposure times. The dependence of sensor emission ratio variability on illumination exposure times between 0.15 and 1.5 s was measured to identify exposure times where spatial variation was minimized (Figure 4A). Spatial variation was highest for exposure times less than 0.4 s. Spatial variation was lowest for exposure times between 0.4 and 0.9 s. An optimal exposure time of 0.8 s was chosen within this range as a duration for the follow up experiments. The red:green ratio for different wells of a 96-well plate for an exposure time of 0.8 s at two levels of oxygenation (0% and 100% air-saturation) and 2 cell culture media (hiPSC-CMs and cardiac fibroblast) varied within ±6% (Figures 4B, C). The standard deviation for hiPSC-CM media was 6% and 2%, for Cal0 and Cal100, respectively; the standard deviation for cardiac fibroblasts media was 3.5% and 1.7% for Cal0 and Cal100, respectively. This variability was much lower than the typical variability between experimental groups of cultured cells.

### 3.5 Temperature effect and time-dependent system calibration

**FIGURE 5:**
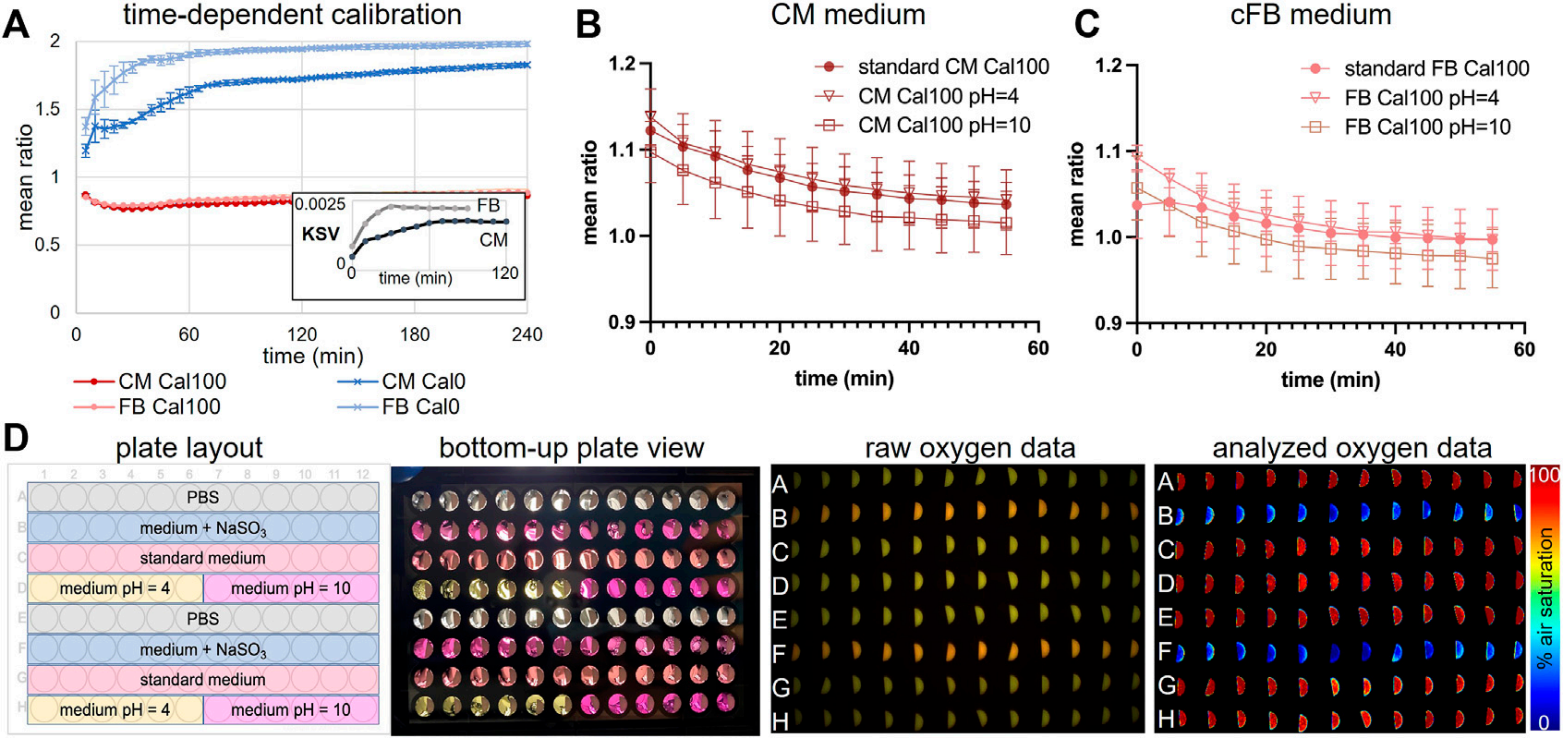
Time-dependent calibration, effects of pH and medium color on the oxygen readings. **(A)**. Time-dependent calibration over 4 h, done in culture medium for human cFBs and human iPSC-CMs in a 37°C cell culture incubator, *n* = 96 wells. Over the first 40–90 min, there is a temperature-dependent rise in the ratio reading for the Cal0 values. Inset shows that the derived Stern-Volmer coefficient, KSV, varies in this initial period for both culture media. **(B)**. Time-dependent calibration changes of CM medium with different pH = 4, 7.4 (standard) and 10. Oxygen readouts were continuously recorded for an hour in 5 min intervals. The mean ratio of air-saturated CM medium with standard and altered pH was plotted over time (mean ± SD, *n* = 12–24 samples). **(C)**. Same as **(B)**, but for cFB culture medium. **(D)**. Full 96-well plate characterization of medium color and pH’s influence on oxygen reading. Conditions are listed in the plate layout. Although pH visibly altered culture medium color (bottom-up view), the analyzed oxygen data were not influenced by medium color or pH difference.

Temperature had a significant effect on the red:green emission ratio for each sensor, which was evident after placing a 96-well plate at room temperature in the incubator maintained at 37°C. The effect of temperature on emission ratio, as a plate was warmed to 37°C, was measured over 4 h after placing a plate in the incubator (Figure 5A). Wells contained media for hiPSC-CMs or media for cardiac fibroblasts, and wells had an oxygen level of either 0% or 100% air-saturation. Oxygen images were acquired every 5 min. The average red:green ratio for each combination of media and oxygenation changed over the first 40–90 min (40 min for the cFB media and 90 min for the hiPSC-CM media) (Figure 5A). Ratios for wells having media at 100% air-saturation were similar throughout the 4 h and changed approximately 10% in the first hour. Ratios for wells having media at 0% changed as much as 60% in the first 2 hours and the change was greater for wells with fibroblast media. This result was likely due to differences in the content of the two types of media. The Stern-Volmer *ksv* values (Eq. 1) computed from the ratios for wells having media at 0% also varied within the first 40–90 min (Figure 5A, inset). These results necessitated the development of a protocol to correct for the effect of changes in well temperature as a plate was warmed to 37°C and for the effect of cell culture media content that determines media color.

### 3.6 Effects of media color and pH

The substrate, salt, and chemical content of the hiPSC-CM media and the cardiac fibroblast media were different, resulting in each culture media having a distinct color. Media pH also determines media color and changes in pH could have an independent effect on sensor luminescence. The effect of media color and pH on sensor emission ratio was studied using 96-well plates and the VisiSensTD system. Wells contained either PBS, hiPSC-CM media, or cardiac fibroblast media and the pH of each well was set to be either 4 or 10 (Figure 5B, plate layout). Differences in the color of the media in each well were clearly visible (Figure 5B, bottom-up plate view). However, color differences were not evident in images of the emission ratio (Figure 5B, raw oxygen data). Color differences were also not evident in pseudocolor images after computing the percent oxygen concentration of each well using the emission ratios (Figure 5B, analyzed oxygen data). To determine if pH had a direct effect on sensor luminescence, the emission ratio of each media having 100% air-saturation at standard pH of 7 or a pH of 4 or 10 was measured once every 5 min for 1 hour using the VisiSensTD system (Figures 5C, D). No significant difference was detected between media having a pH of 7 and 4. The emission ratio was consistently lower for media having a pH of 10, which is highly alkaline and has less biological relevance for cell culture experiments.

### 3.7 Long-term monitoring of peri-cellular oxygen concentration in cardiac cells

**FIGURE 6:**
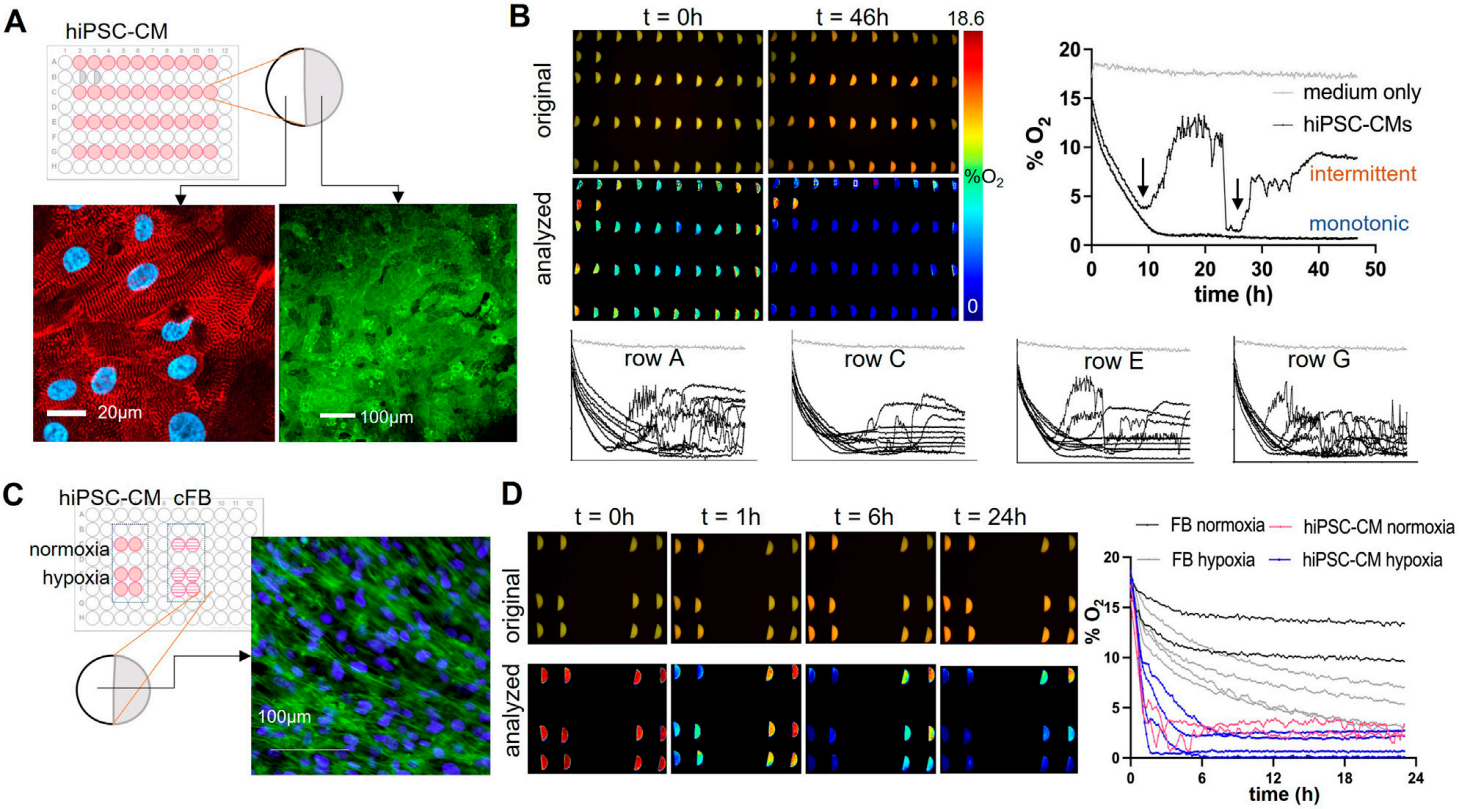
Optical measurements of peri-cellular oxygen in human iPSC-cardiomyocytes and human cardiac fibroblasts, cFBs. **(A)**. Full plate hiPSC-CM oxygen measurement plate layout and still images of hiPSC-CMs grown on glass bottom half of the well (upper image) and on the oxygen sensor (lower image). In the upper image, the hiPSC-CMs grown on glass were antibody-labeled for alpha-actinin (red), nuclei were labeled using Hoechst dye (blue). The lower image of hiPSC-CMs growing on top of the oxygen sensors, were collected using an upright microscope with a water immersion lens; the eYFP signal from channelrhodopsin2-eYFP infected cardiomyocytes is shown. **(B)**. Continuous optical sensing of peri-cellular O_2_ in human iPSC-CMs over 48 h. Peri-cellular oxygen levels dropped to <5% in <10 h for most samples. Two types of hypoxia responses were seen: (1) monotonic O_2_ depletion to a steady state, and (2) intermittent O_2_ dynamics after an O_2_ dip below 5% (black arrows). Type 1 was observed in about 30% of the samples in this plate, while type 2 was observed in the remaining 70% of the measured *n* = 40 wells. The specific type of response was independent of spatial location—traces from the four different rows in the plate having samples are shown (rows A, C, E, G). 18.6% oxygenation corresponds to the maximum (100%) oxygen saturation in a CO_2_ incubator. **(C)**. Experimental design of hiPSC-CM and cFBs hypoxia study, and still image of cFBs on the glass bottom portion of a well. F-actin (green) highlights the cytoskeleton of the cells, and nuclei (Hoechst labeled) are indicated in blue. **(D)**. Example images and calibrated traces of hiPSC-CMs and cFB peri-cellular O_2_ over 24h, under normoxic and hypoxic conditions. Peri-cellular O_2_ in cFBs decreased slower and stabilized to a higher steady-state level; induced hypoxia (filling the wells with solution to the top and sealing them) sped up the O_2_ depletion in cFB. The hiPSC-CMs showed rapid depletion of O_2_ within 6 h with a faster initial phase of drop in the sealed samples, which then bounced back to slightly higher steady-state values.

After validation, characterization, and the development of a robust calibration protocol, the VisiSensTD oxygen imaging system was used to continuously measure peri-cellular oxygen concentration from cardiac cells cultured in a 96-well plate. Using an upright microscope with a water-immersion objective, we confirmed that human iPSC-CMs formed a dense cellular syncytium on top of the oxygen sensors after coating with fibronectin (Figure 6A, lower image). The transparent half of the glass-bottom of each well that was not covered by a sensor enabled human iPSC-CMs and human cardiac fibroblasts to be monitored to confirm healthy growth. This corroborated no adverse effects of the sensors as cells thrived as a syncytium equally well on top of each sensor and on the glass-bottom portion of the well that was not covered by a sensor. In monitoring cell function over several days, we confirmed that sensor readouts were stable and did not alter cell growth. Typical images of human iPSC-CMs and human cFBs grown on fibronectin-coated glass-bottom portion are shown in Figure 6A, upper image and Figure 6C, respectively. Studies were conducted for time intervals ranging from 24 to 48 h (Figure 6). In one experiment, peri-cellular oxygen was measured every 10 min over 48 h from hiPSC-CMs plated in every other row of a 96-well plate (Figure 6A). Two control wells had oxygen sensors and media but no cells. Peri-cellular oxygen dropped dramatically to <5% within 10 h in all wells that had cells. Oxygen concentration in control wells was constant at 18.6%, corresponding to the equilibration of the media with the incubator oxygen concentration. In wells having cells, two patterns of peri-cellular oxygen dynamics emerged after 10 h (Figure 6B): one of monotonic oxygen depletion and another of intermittent depletion. In cases of monotonic depletion, after the initial oxygen drop to <5%, peri-cellular oxygen monotonically and slowly decreased to a steady-state level. In intermittent depletion, peri-cellular oxygen oscillated intermittently after the initial oxygen drop. For the results shown in Figure 6B, monotonic depletion was observed in 11 out of 38 wells (29%) and intermittent depletion occurred in 27 out of 38 wells (71%). Oscillatory intermittent depletion occurred in different wells within the rows of a plate, confirming that oxygen dynamics were independent of the position of a well within a plate.

Dynamic patterns of peri-cellular oxygen were studied in cardiac fibroblast and hiPSC-CM cultures over 24 h to determine if depletion occurs more rapidly in cells having higher metabolic rate (Figures 6C, D). Depletion was hypothesized to occur more rapidly in hiPSC-CM cultures due to the higher oxygen consumption of contracting cells. The two cell types were cultured in separate sets of six wells of the same plate. Two of those wells were filled with the typical 200 ml of media to provide normoxia while the other four wells were filled with 300 ml of media and sealed with oxygen-impermeable tape to generate hypoxia (Figure 6C). Peri-cellular oxygen was depleted within 6 h for normoxic and hypoxic cultures of hiPSC-CMs. Oxygen depletion was much slower for fibroblasts, where over 24 h oxygen did not drop below 10% for normoxic cultures and most hypoxic cultures maintained an oxygen level above 5% (Figure 6D). These results confirm the hypothesis and also demonstrate the utility of optical oxygen sensing in providing long-term measurements that reveal cell-type differences in the baseline and fluctuations of peri-cellular oxygen concentration.

## 4 Discussion

Oxygen consumption and defense mechanisms against hypo-/hyperoxia are critical to sustaining life, as O_2_ is a key component of energy (ATP) production in the mitochondria. However, when in excess, oxygen is inherently toxic due to its chemical reactivity and the generation of reactive oxygen species (ROS) (Keeley and Mann, 2019). Although the need for measuring peri-cellular oxygen levels in cell culture has been recognized since at least 1970 (Keeley and Mann, 2019), doing so is not routine in cell culture studies.

Recent developments have included scalable solutions to control dissolved (bulk) oxygen levels in custom-designed 96-well plates. For example, in one study the well bottoms of a 96-well plate were modified to use special actuation posts, driven by magnetic field to increase oxygen availability (Fisher et al., 2021). In other studies, elegant microfluidics-based solutions with concentration gradients have been deployed to control dissolved oxygen levels in custom-designed 96-well plates with oxygen-permeable bottom (Yao et al., 2018; Yao et al., 2021). Fiber-optic probe was used to perform sequential measurements in different wells and confirm oxygen levels close to the well bottom, which for cells with low metabolic needs may approach peri-cellular oxygen levels.

In bacterial cell culture applications, more advanced high-throughput monitoring solutions have been implemented. For example, oxygen-sensing fluorophores have been embedded in hydrogel inserts to monitor dissolved oxygen levels in custom 96-well plates up to several hours (Wu et al., 2018). Another bacterial cell culture study monitored peri-cellular oxygen by methods similar to the ones reported here, including the use of a commercial 96-well OxoPlate by Presens (Glauche et al., 2015). In one variant, circular sensor patches were cut and glued in standard 96-well plates; a robotic arm was moving the plate between a shaker and a plate reader to track oxygen levels as function of shaking speeds to optimize microbial culture conditions. Because of the discontinuous readouts, the study purposefully sought to develop oxygen sensors with slower response time than the ones deployed here. OxoPlate (Presens) was also used to study antibiotic resistance in microbial cell culture based on longitudinal tracking of peri-cellular oxygen (Almatrood et al., 2022).

Overall, high-throughput peri-cellular oxygen measurements in mammalian cell culture are rare, and no long-term measurements are available from cultures of human iPSC-CMs. A major obstacle has been the lack of user-friendly monitoring systems and reliable non-invasive methods for long-term oxygen monitoring in common cell culture formats such as 96-well plates.

In this study, the Presens ratiometric optical oxygen sensors and camera-based imaging system were adopted to track peri-cellular oxygen dynamics in human cardiac cells cultured in glass-bottom 96-well plates. We developed a scalable technique for reproducible cutting and positioning of semicircular optical oxygen sensors into glass-bottom HT plates (Figure 1), to cover half of each well, leaving the other half for multimodal structural and functional imaging. We validated the optical oxygen readings with traditional Clark electrodes positioned in line in bulk measurements and during perfusion within 96 wells using our specialized microfluidics-based cover. The spectral, spatial, and temporal properties of the system were systematically characterized (Figures 2–4). Optimization of the exposure time and the two-point calibration (Figures 4, 5) allowed continuous oxygen measurements in a standard incubator that were robust and reliable.

Compared to prior studies, the following new and useful aspects are demonstrated here: 1) to our knowledge, this is the first mammalian culture study to track long-term peri-cellular oxygen in standard glass-bottom 96-well plates, that are the industry standard and are used in preclinical testing; 2) the first study to do so in human cardiomyocytes over long term, with continuous frequent sampling of peri-cellular oxygen levels during cell growth in a standard cell culture incubator, providing novel insights on cardiomyocyte oxygen consumption dynamics; 3) our approach in using half of each well for optical oxygen sensing (semicircular sensors) allows multimodal structural and functional observations in the same samples through the transparent glass bottom part, compatible with standard microscopy and with all-optical electrophysiology systems.

Our results are in agreement with *in vivo* estimates of peri-cellular oxygen levels in the heart, where oxygen tension in the immediate vicinity of the cardiomyocytes is in the range of 3%–6% (Keeley and Mann, 2019). Namely, for human iPSC-CMs, in the static culture conditions of a glass-bottom 96-well plate, we observed a drop to <5% oxygen within several hours of media exchange. After this initial drop, the cells either settled to a steady state of low pericellular oxygen, likely due to the balance between active oxygen consumption and passive diffusion, or exhibited an intermittent oxygen dynamics (Figure 6). These two types of responses are interesting novel observations and require further investigation. In contrast to the cardiomyocytes, the non-contracting cardiac fibroblasts exhibited higher peri-cellular oxygen levels after dropping to a stable steady-state, as expected for cells with lower oxygen demand.

*In vivo*, cardiac cell normoxia is maintained by feedback mechanisms that tightly regulate coronary blood (Hoffman and Buckberg, 2014) and by oxygen-on-demand provided by the excellent oxygen carrying capacity of hemoglobin. *Ex vivo*, in the absence of adequate oxygen buffering, perfused working hearts experience hypoxic conditions (Garrott et al., 2017; Kuzmiak-Glancy et al., 2018). *In vitro*, cardiomyocytes may experience hyperoxic, normoxic or hypoxic conditions (Keeley and Mann, 2019) depending on their density, electromechanical activity, mass transport conditions and the shortest path to the ambient oxygen supply. For human iPSC-CMs, oxygen tension was recognized in early work as a key variable for optimizing cellular differentiation and maturation; transient control of oxygen level is used during the differentiation of iPS cells into cardiomyocytes (Lian et al., 2015; Lyra-Leite et al., 2022). Hypoxia signaling is also a foundational physiologic component of mature cardiomyocytes, where it intimately regulates electromechanical function, including ion channel currents and protein expression (Keeley et al., 2018; Keeley and Mann, 2019; Pressler et al., 2022). Based on this, longitudinal label-free monitoring of peri-cellular oxygen, in a high-throughput manner, provides unique insights into the metabolic state of the cells, and potentially can be correlated with their level of maturation.

The system described here is best suited for imaging the oxygen concentration of two-dimensional multi-cell structures, such as monolayers. This is a limitation, considering the growing popularity of three-dimensional cell constructs, including cell spheroids and microtissues. A potential way to extend the described label-free oxygen sensing approach to 3D structures is inspired by the recent work of others, where optical sensors have been mounted on transparent prisms (Peniche Silva et al., 2020) and oxygen-sensing cell culture vessels have been thermoform-molded to line the wells of spheroidal plates (Grün et al., 2023). Future work includes automating the placement of sensors into 96-well plates using the scalable technique described and manually demonstrated here. Combination of the high-throughput monitoring of peri-cellular oxygen with microfluidics-based solutions of controlling oxygenation (Wei et al., 2020; Yao et al., 2021) can yield feedback-controlled growth environment for cardiac tissue engineering. Coupling label-free measurements of peri-cellular oxygen with label-free measurements of cardiac electromechanical waves (Liu et al., 2023) will also provide valuable insights into the interplay between cellular activity and oxygenation state.

## Data Availability

The original contributions presented in the study are included in the article/Supplementary Material, further inquiries can be directed to the corresponding authors.
